# Plant Preparations and Compounds with Activities against Biofilms Formed by *Candida* spp.

**DOI:** 10.3390/jof7050360

**Published:** 2021-05-05

**Authors:** Tomasz M. Karpiński, Marcin Ożarowski, Agnieszka Seremak-Mrozikiewicz, Hubert Wolski, Artur Adamczak

**Affiliations:** 1 Department of Medical Microbiology, Poznań University of Medical Sciences, Wieniawskiego 3, 61-712 Poznań, Poland; 2 Department of Biotechnology, Institute of Natural Fibres and Medicinal Plants, National Research Institute, Wojska Polskiego 71b, 60-630 Poznań, Poland; marcin.ozarowski@iwnirz.pl; 3 Division of Perinatology and Women’s Diseases, Poznań University of Medical Sciences, Polna 33, 60-535 Poznań, Poland; asm@data.pl (A.S.-M.); hubertwolski@wp.pl (H.W.); 4 Laboratory of Molecular Biology in Division of Perinatology and Women’s Diseases, Poznań University of Medical Sciences, Polna 33, 60-535 Poznań, Poland; 5 Department of Pharmacology and Phytochemistry, Institute of Natural Fibres and Medicinal Plants, National Research Institute, Kolejowa 2, 62-064 Plewiska, Poland; 6 Division of Gynecology and Obstetrics, Podhale Multidisciplinary Hospital, Szpitalna 14, 34-400 Nowy Targ, Poland; 7 Department of Botany, Breeding and Agricultural Technology of Medicinal Plants, Institute of Natural Fibres and Medicinal Plants, National Research Institute, Kolejowa 2, 62-064 Plewiska, Poland; artur.adamczak@iwnirz.pl

**Keywords:** *Candida*, biofilm, treatment, antifungals, natural compounds, essential oil, extract, minimal inhibitory concentration (MIC)

## Abstract

Fungi from the genus *Candida* are very important human and animal pathogens. Many strains can produce biofilms, which inhibit the activity of antifungal drugs and increase the tolerance or resistance to them as well. Clinically, this process leads to persistent infections and increased mortality. Today, many *Candida* species are resistant to drugs, including *C. auris*, which is a multiresistant pathogen. Natural compounds may potentially be used to combat multiresistant and biofilm-forming strains. The aim of this review was to present plant-derived preparations and compounds that inhibit *Candida* biofilm formation by at least 50%. A total of 29 essential oils and 16 plant extracts demonstrate activity against *Candida* biofilms, with the following families predominating: Lamiaceae, Myrtaceae, Asteraceae, Fabaceae, and Apiacae. *Lavandula dentata* (0.045–0.07 mg/L), *Satureja macrosiphon* (0.06–8 mg/L), and *Ziziphora tenuior* (2.5 mg/L) have the best antifungal activity. High efficacy has also been observed with *Artemisia judaica*, *Lawsonia inermis*, and *Thymus vulgaris*. Moreover, 69 plant compounds demonstrate activity against *Candida* biofilms. Activity in concentrations below 16 mg/L was observed with phenolic compounds (thymol, pterostilbene, and eugenol), sesquiterpene derivatives (warburganal, polygodial, and ivalin), chalconoid (lichochalcone A), steroidal saponin (dioscin), flavonoid (baicalein), alkaloids (waltheriones), macrocyclic bisbibenzyl (riccardin D), and cannabinoid (cannabidiol). The above compounds act on biofilm formation and/or mature biofilms. In summary, plant preparations and compounds exhibit anti-biofilm activity against *Candida*. Given this, they may be a promising alternative to antifungal drugs.

## 1. Introduction

The genus *Candida* contains about 150 species; however, most are environmental organisms. The most medically important is *Candida albicans*, which accounts for about 80% of infections. *C. albicans* causes more than 400,000 cases of bloodstream life-threatening infections annually, with a mortality rate of about 42% [1]. *Candida* non-*albicans* species that are mainly responsible for infections are *C. glabrata*, *C. parapsilosis*, *C. tropicalis*, *C. krusei*, and *C. dubliniensis* [2]. Less frequently identified are *C. guilliermondii*, *C. lusitaniae*, *C. rugosa*, *C. orthopsilosis*, *C. metapsilosis*, *C. famata*, *C. inconspicua*, and *C. kefyr* [3].

*C. albicans* is a member of the commensal microflora. It colonizes the oral mucosal surface of 30–50% of healthy people. The rate of carriage increases with age and in persons with dental prostheses up to 60% [4,5,6]. Opportunistic infection caused by *Candida* species is termed candidiasis. At least one episode of vulvovaginal candidiasis (or thrush) concerns 50 to 75% of women of childbearing age [7]. Candidiasis can also affect the oral cavity, penis, skin, nails, cornea, and other parts of the body. In immunocompromised persons, untreated candidiasis poses the risk of systemic infection and fungemia [5,8]. *Candida* can be an important etiological factor in the infection of chronic wounds that are difficult to treat; this is mainly related to the production of biofilm [9].

Treatment of candidiasis depends on the infection site and the patient’s condition. According to guidelines, vulvovaginal candidiasis should be treated with oral or topical fluconazole; however, regarding *C. glabrata* infection, topical boric acid, nystatin, or flucytosine is suggested. In oropharyngeal candidiasis, the treatment options include clotrimazole, miconazole, or nystatin, and in severe disease, fluconazole or voriconazole. In candidemia and invasive candidiasis, the drugs of choice are echinocandins (caspofungin, micafungin, anidulafungin), fluconazole, or voriconazole; in resistant strains, amphoteticin B is used. In selected cases of candidemia caused by *C. krusei*, voriconazole is recommended [10,11,12]. More details can be found in the Guidelines of the Infectious Diseases Society of America [12] and the European Society of Clinical Microbiology and Infectious Diseases [11]. Increasingly, *Candida* species are becoming resistant to drugs. Marak and Dhanashree [13] tested the resistance of 90 *Candida* strains isolated from different clinical samples, such as pus, urine, blood, and body fluid. Their study revealed that about 41% of *C. albicans* strains are resistant to fluconazole and voriconazole. Simultaneously, about 41% of *C. tropicalis* strains are resistant to voriconazole and about 36% of strains to fluconazole. In strains of *C. krusei*, about 23% are resistant to fluconazole and about 18% to voriconazole. Rudramurthy et al. [14] studied resistance in *C. auris*, which is considered a multiresistant pathogen. Among 74 strains obtained from patients with candidemia, over 90% of strains were resistant to fluconazole and about 73% to voriconazole. Virulence factors of *Candida* species include the secretion of hydrolases, the transition of yeast to hyphae, phenotypic switching, and biofilm formation [15,16]. All microorganisms in biofilm form are more resistant to antimicrobial and host factors, which leads to difficulties in eradication [17]. It has also been shown that resistance to drugs increases significantly in the case of *Candida* biofilm occurrence. Biofilm prevents the spread of antifungals; moreover, fluconazole is bound by the biofilm matrix [18]. The formation of a *Candida* biofilm during infection increases mortality, length of hospital stay, and cost of antifungal therapy [19].

Due to the above, new antifungal drugs are sought that could effectively combat not only planktonic fungi but also fungal biofilms. The natural compounds offer promise, with many acting on *Candida* species or biofilms in vitro [20].

The aim of this review was to present plant-derived natural compounds that have an effect against biofilms formed by *Candida* species.

## 2. Materials and Methods 

In this review, publications available in PubMed and Scopus databases and through the Google search engine were taken into account. The following keywords and their combinations were used: “antifungal,” “Candida,” “anti-biofilm,” “biofilm,” “plant,” “compound,” “extract,” and “essential oil.” The principal inclusion criterion was the inhibition of biofilm formation by at least 50%. We focused on biofilm inhibition assays, in which the time of culture allowed for *Candida* biofilm maturation was at least 24 hours. Articles from the year 2000 to the present were taken into account. All articles published in predatory journals were rejected.

## 3. Results and Discussion

### 3.1. Plant Preparations That Display Activity against Candida Biofilms

The present review includes 60 articles in which *Candida* biofilm formation was inhibited by at least 50%. It has been shown that preparations from 34 plants demonstrate activity against *Candida* biofilms. Among them were 29 essential oils and 16 extracts. The plants from the following families dominated: Lamiaceae (6 species in 5 genera), Myrtaceae (5 species in 4 genera), Asteraceae (4 species in 4 genera), Fabaceae (4 species in 3 genera), and Apiacae (4 species in 2 genera). 

Plants from the Lamiaceae family had the best antifungal activity, including *Lavandula dentata* (0.045–0.07 mg/L) [21], *Satureja macrosiphon* (0.06–8 mg/L) [22], and *Ziziphora tenuior* (2.5 mg/L) [23]. *Artemisia judaica* (2.5 mg/L) from the Asteraceae family [24], *Lawsonia inermis* (2.5–12.5 mg/L) from the Lythraceae family [25], and *Thymus vulgaris* (12.5 mg/L) from the Lamiaceae family [26] likewise exhibited good antifungal activity (Table 1). All preparations were essential oils, with the exception of *Lawsonia inermis*, which was an extract. Most of the plant preparations presented in Table 1 acted on biofilm formation and/or mature biofilms.

Antibiofilm activity may vary between plants in the same family. For example, in the Lamiaceae family, essential oil from *Lavandula dentata* acted against *C. albicans* biofilm at concentrations of 0.045–0.07 µL/mL [21], while essential oil from *Satureja hortensis* acted against the same biofilm at concentrations of 400–4800 mg/L [51]. There may also be large differences within the same species, due to various reasons. This may be influenced by, for example, different research methodologies, the use of different strains of fungi, and different chemical compositions depending on the plant variety, country, and season of harvest. A notable example of such a difference is observed with *Mentha × piperita*. In studies by Benzaid et al. [44], essential oil of *M. piperita* acted against *Candida* biofilm at a concentration of 10 µL/mL. However, the work of Agarwal et al. [38] showed that the same essential oil was active at 800 µL/mL.

Changes in the content of active substances were described by Gonçalves et al. [56]. They showed that in essential oil from *Mentha cervina* collected in August, the amount of isomenthone was 8.7% and pulegone was 75.1%. However, in essential oil collected in February, the ratio of the two compounds reversed and amounted to 77.0% for isomenthone and 12.9% for pulegone. The method of obtaining the compounds likewise had an influence on their content in the final essential oil. In a study by Ćavar et al. [57], the composition of essential oils of *Calamintha glandulosa* differed depending on the extraction method. The level of menthone was 3.3% using aqueous reflux extraction, 4.7% using hydrodistillation, and 8.3% using steam distillation, while the concentration of shisofuran was only 0.1% using hydrodistillation and steam distillation, while aqueous reflux yielded 9.7%. 

### 3.2. Plant Compounds That Display Activity against Candida Biofilm

It has been shown that 69 compounds obtained from plants demonstrate activity against *Candida* biofilms (Table 2). Among these, the most common are monotherpenes (20), followed by sesquiterpene lactones (7) and sesquiterpenes (6). Another big group is also phenolic compounds, including phenols (6), phenolic acids (5), phenolic aldehydes (2), polyphenols (2), and phenolic alcohol (1). 

In terms of activity, large differences were found, depending on the authors cited. Eugenol and thymol serve as good examples. Both compounds exhibited excellent activity in some studies (from 12.5 mg/L for eugenol [58] and 1.56 mg/L for thymol [26]), and in other studies, the activity was very poor (up to 80,000 for both [59]). These differences may be related, for example, to a different purity of the compound, a different fungal suspension density, or even to the use of other *Candida* strains with different sensitivities to chemical substances. A number of other factors, such as the type of culture medium, pH of the medium, incubation time, and temperature may likewise influence the antimicrobial activity [20].

According to the European Committee on Antimicrobial Susceptibility Testing (EUCAST), the antifungal clinical breakpoints are between 0.001 mg/L and 16 mg/L [60]. Using EUCAST guidelines in this review, the most active compounds that inhibit (>50%) *Candida* biofilm formation are lichochalcone A (from 0.2 mg/L) [61], thymol (from 3.12 mg/L) [26], dioscin (from 3.9 mg/L) [31], baicalein (from 4 mg/L) [62], warburganal (4.5 mg/L) [52], pterostilbene, waltheriones and riccardin D (both from 8 mg/L) [63,64,65], polygodial (10.8 mg/L) [52], cannabidiol and eugenol (both from 12.5 mg/L) [58,66], and ivalin (15.4 mg/L) [67]. It is interesting that monotherpenes, which represent the highest percentage of substances listed in Table 2, are not the most active compounds. The two larger groups with the best activity are phenolic compounds (thymol, pterostilbene, and eugenol), and sesquiterpene derivatives (warburganal, polygodial, and ivalin). Single compounds with the highest observed activity belong to chalconoids (lichochalcone A), steroidal saponins (dioscin), flavonoids (baicalein), alkaloids (waltheriones), macrocyclic bisbibenzyls (riccardin D), and cannabinoids (cannabidiol). Most of the compounds presented in Table 2 acted on biofilm formation and/or mature biofilm.

## 4. Conclusions

Plant preparations (essential oils and extracts) and pure compounds exhibit anti-biofilm activity against *Candida* species. Some of them are characterized by high activity in concentrations below 16 mg/L. Given this activity at relatively low concentrations, some may prove to be promising alternatives to antifungal drugs, especially in the cases of resistant or multiresistant strains of *Candida*. Moreover, the simple chemical structures involved and relative ease of extraction from natural sources warrant further research into the development of new, promising, and much-needed plant-based antifungals.

## Figures and Tables

**Table 1:** Antifungal (MICs) and anti-biofilm (inhibition >50%) activity of plant preparations (essential oils or extracts).

Name of Plant (Family)	Main Compounds Presented in the Reference (EO: Essential Oil)	Targeted Species of *Candida*	MICs (mg/L; mL/L)	Inhibition of Biofilm Formation by at Least 50% (mg/L; mL/L)	Inhibited Stage of Biofilm; Method of Biofilm Detection	Ref.
*Acorus calamus var. angustatus* Besser = *A. tatarinowii* Schott (Acoraceae)	EO: asaraldehyde, 1-(2,4,5-trimethoxyphenyl)-1,2-propanediol, α-asarone, β-asarone, γ-asarone, acotatarone C	*C. albicans*	51.2	50–200	Mature biofilm; crystal violet and fluorescence microscopy	[27]
*Allium sativum* L. (Amaryllidaceae)	Extract: allicin	*C. albicans*	400	60	Biofilm formation; XTT	[28]
*Aloysia gratissima* (Aff & Hook).Tr (Verbenaceae)	EO: E-pinocamphone (16.07%), β-pinene (12.01%), guaiol (8.53%), E-pinocarveol acetate (8.19%)	*C. albicans*	15	500	Biofilm formation; crystal violet	[29]
*Artemisia judaica* L. (Asteraceae)	EO: piperitone (30.4%), camphor (16.1%), ethyl cinnamate (11.0%), chrysanthenone (6.7%)	*C. albicans*	1.25	2.5	Mature biofilm; XTT	[24]
		*C. guillermondii*	1.25	2.5		
		*C. krusei*	1.25	2.5		
		*C. parapsilosis*	1.25	2.5		
		*C. tropicalis*	1.25	2.5		
*Buchenavia tomentosa* Eichler (Combretaceae)	Extract: gallic acid, kaempferol, epicatechin, ellagic acid, vitexin, and corilagin	*C. albicans*	625	312.5	Biofilm formation and mature biofilm; culture	[30]
*Chamaecostus cuspidatus* (Nees & Mart.) C.Specht & D.W.Stev. (Costaceae)	Extract: dioscin, aferoside A, aferoside C	*C. albicans*	250	15.62	Biofilm formation and mature biofilm; MTT	[31]
*Cinnamomum verum* J. Presl (Lauraceae)	EO: eugenol (77.22%), benzyl benzoate (4.53%), *trans*-caryophyllene (3.39%), acetyl eugenol (2.75%), linalool 2.11%	*C. albicans*	1000	150	Biofilm adhesion; XTT	[32]
		*C. dubliniensis*	1000	200		
		*C. tropicalis*	1000	350		
*Citrus limon* (L.) Osbeck (Rutaceae)	EO: limonene (53.4%), neral (11%), geraniol (9%), *trans*-limonene oxide (7%), nerol (6%)	*C. albicans*	500	2000	Biofilm formation and mature biofilm; XTT	[33]
		*C. glabrata*	250	1000		
		*C. krusei*	500	125		
		*C. orthopsilosis*	500	1000		
		*C. parapsilosis*	500	2000		
		*C. tropicalis*	250	2000		
*Copaifera paupera* (Herzog) Dwyer (Fabaceae)	Extract: galloylquinic acids, quercetrin, afzelin	*C. glabrata*	5.89	46.87	Biofilm formation and mature biofilm; XTT	[34]
*Copaifera reticulata* Ducke (Fabaceae)	Extract: galloylquinic acids, quercetrin, afzelin	*C. glabrata*	5.89	46.87	Biofilm formation and mature biofilm; XTT	[34]
*Coriandrum sativum* L. (Apiaceae)	EO: 1-decanol (33.91%), E-2-decen-1-ol (23.59%), 2-dodecen-1-ol (13.06%), E-2-tetradecen-1-ol (5.46%)	*C. albicans*	7	250	Biofilm formation; crystal violet	[29]
	EO: decanal (19.09%), *trans*-2-decenal (17.54%), 2-decen-1-ol (12.33%), cyclodecane (12.15%)	*C. albicans*	15.6	62.5–125	Biofilm adhesion; crystal violet	[35]
		*C. dubliniensis*	31.2	62.5–125		
		*C. rugosa*	15.6	62.5		
		*C. tropicalis*	31.2	31.25–250		
*Croton eluteria* (L.) W.Wright (Euphorbiaceae)	EO: α-pinene (29.37%), β-pinene (19.35%), camphene (10.31%), 1,8-cineole (9.68%)	*C. albicans*	4000	5–500	Biofilm formation; confocal laser microscopy	[36]
*Cupressus sempervirens* L. (Cupressaceae)	EO: sabinene (20.3%), citral (20%), terpinene-4-ol (15.4%), α-pinene (8%)	*C. albicans*	250	1000	Biofilm formation and mature biofilm; XTT	[33]
		*C. glabrata*	31.25	250		
		*C. krusei*	62.5	62.5		
		*C. orthopsilosis*	31.25	125		
		*C. parapsilosis*	62.5	500		
		*C. tropicalis*	250	500		
*Cymbopogon citratus* (DC.) Stapf (Poaceae)	EO: no composition	*C. albicans*	180–360	22.5–180	Biofilm formation; XTT	[37]
*Cymbopogon martini* (Roxb.) W.Watson (Poaceae)	EO: no composition	*C. albicans*	16,800	800	Biofilm formation; XTT	[38]
*Cymbopogon nardus* (L.) Rendle (Poaceae)	EO: citronellal (27.87%), geraniol (22.77%), geranial (14.54%), citronellol (11.85%), neral (11.21%)	*C. albicans*	1000	2500–5000	Biofilm adhesion; XTT	[39]
		*C. krusei*	250–500	2500		
		*C. parapsilosis*	500–1000	5000–10,000		
*Cyperus articulatus* L. (Cyperaceae)	EO: α-pinene (5.72%), mustakone (5.66%), α-bulnesene (5.02%), α-copaene (4.97%)	*C. albicans*	125	250	Biofilm formation; crystal violet	[29]
*Eucalyptus* sp. (Myrtaceae)	EO: no composition	*C. albicans*	8	8	Mature biofilm; luminescence	[40]
*Eucalyptus globulus* Labill. (Myrtaceae)	EO: 1,8-cineole (75.8%), p-cymene (7.5%), α-pinene (7.4%), limonene (6.4%)	*C. albicans*	219	11,250–22,500	Mature biofilm; atomic force microscopy	[41]
		*C. glabrata*	219	11,250–22,500		
		*C. tropicalis*	885	11,250–22,500		
	EO: no composition	*C. albicans*	8400	500	Biofilm formation; XTT	[38]
*Eugenia brasiliensis* Lam. (Myrtaceae)	Extract: no composition	*C. albicans*	15.62–31.25	156	Mature biofilm; scanning electron microscopy	[42]
*Eugenia leitonii* Legrand *nom. inval.* (Myrtaceae)	Extract: no composition	*C. albicans*	15.62–250	156	Mature biofilm; scanning electron microscopy	[42]
*Helichrysum italicum* (Roth) G.Don (Asteraceae)	EO: α-pinene (27.64%), γ-elemene (23.84%), β-caryophyllene (13.05%), α-longipinene (11.25%)	*C. albicans*	6000	10–500	Biofilm formation; confocal laser microscopy	[36]
*Laserpitium latifolium* L. (Apiaceae)	Extract: laserpitine	*C. albicans*	1250	6300	Mature biofilm; luminescence	[43]
		*C. krusei*	1250	6300		
*Laserpitium ochridanum* Micevski (Apiaceae)	Extract: isomontanolide, montanolide, tarolide	*C. albicans*	5000	10,000	Mature biofilm; luminescence	[43]
		*C. krusei*	5000	10,000		
*Laserpitium zernyi* Hayek *= L. siler* subsp. *zernyi* (Hayek) Tutin (Apiaceae)	Extract: isomontanolide, montanolide, tarolide	*C. albicans*	7500	15,000	Mature biofilm; luminescence	[43]
		*C. krusei*	7500	37,500		
*Lavandula dentata* L. (Lamiaceae)	EO: eucalyptol (42.66%), β-pinene (8.59%), *trans*-α-bisabolene (6.34%), pinocarveol (6.3%)	*C. albicans*	0.15–0.18	0.045–0.07	Mature biofilm; XTT	[21]
*Lawsonia inermis* L. (Lythraceae)	Extract: no composition	*C. albicans*	10	2.5–12.5	Mature biofilm; MTT	[25]
*Lippia sidoides* Cham. (Verbenaceae)	EO: thymol (65.76%), p-cymene (17.28%), α-caryophyllene (10.46%), cyclohexanone (6.5%)	*C. albicans*	250	500	Biofilm formation; crystal violet	[29]
*Litsea cubeba* (Lour.) Pers. (Lauraceae)	EO: limonene (37%), neral (31.4%), citral (12%), linalool (4%)	*C. albicans*	500	2000	Biofilm formation and mature biofilm; XTT	[33]
		*C. glabrata*	250	2000		
		*C. krusei*	62.5	250		
		*C. orthopsilosis*	250	2000		
		*C. parapsilosis*	500	1000		
		*C. tropicalis*	1000	2000		
*Mentha × piperita* L. (Lamiaceae)	EO: menthol (32.93%), menthone (24.41%), 1,8-cineole (7.89%)	*C. albicans*	1–10	10	Biofilm formation; MTT	[44]
	EO: no composition	*C. albicans*	11,600	800	Biofilm formation; XTT	[38]
*Mikania glomerata* Spreng (Asteraceae)	EO: germacrene D (38.29%), α-caryophyllene (9.49%), bicyclogermacrene (7.98%), caryophyllene oxide (4.28%)	*C. albicans*	250	500	Biofilm formation; crystal violet	[29]
*Myrtus communis* L. (Myrtaceae)	EO: α-pinene (39.8%), 1,8-cineole (24.8%), limonene (10.7%), linalool (6.4%)	*C. albicans*	1250–10,000	None or 1250	No data; no data	[45]
		*C. parapsilosis*	1250 to >16,000	1250		
		*C. tropicalis*	1250–16,000	1250		
*Ononis spinosa* L. (Fabaceae)	Extract: kaempherol-O-dihexoside, kaempherol-O-hexoside-pentoside, kaempherol-O-hexoside, quercetin-O-hexoside-pentoside, acetylquercetin-O-hexoside	*C. albicans*	620	10,000	Mature biofilm; luminescence	[46]
		*C. krusei*	620	5000		
		*C. tropicalis*	310	10,000		
*Pelargonium graveolens* L’Hér. (Geraniaceae)	EO: geraniol (42.3%), linalool (20.1%), citronellol (11.1%), menthone (8.0%)	*C. albicans*	125	4000–8000	Mature biofilm; XTT	[47]
*Piper claussenianum* (Miq.) C. DC. (Piperaceae)	EO: nerolidols	*C. albicans*	4100–9600	2400–12,600	Mature biofilm; MTT	[48]
*Portulaca oleracea* L. (Portulacaceae)	Extract: no composition	*C. albicans*	10	12.5	Mature biofilm; MTT	[25]
*Punica granatum* L. (Lythraceae)	Extract: ellagic acid	*C. albicans*	1000	100–750	Biofilm formation and mature biofilm; crystal violet	[49]
*Santolina impressa* Hoffmanns. & Link (Asteraceae)	EO: β-pinene (22.5%), 1,8-cineole (10.0%), limonene (9.1%), camphor (8.1%), β-phellandrene (8.0%)	*C. albicans*	540	70–1050	Biofilm formation; XTT	[50]
*Satureja hortensis* L. (Lamiaceae)	EO: thymol (45.9%), gamma-terpinen (16.71%), carvacrol (12.81%), p-cymene (9.61%)	*C. albicans*	200–400	400–4800	Biofilm adhesion, formation, and mature biofilm; MTT	[51]
*Satureja macrosiphon* (Coss.) *= Micromeria macrosiphon* Coss. (Lamiaceae)	EO: linalool (28.46%), borneol (16.22%), terpinene-4-ol (14.58%), *cis*-sabinene hydrate (12.96%)	*C. albicans*	0.06–4	0.06–8	Biofilm formation; XTT	[22]
		*C. dubliniensis*	0.25–4	2–8		
*Syzygium aromaticum* (L.) Merr. & L.M.Perry *= Eugenia caryophyllus* (Spreng.) Bullock & S.G.Harrison (Myrtaceae)	EO: no composition	*C. albicans*	100–200	50	Biofilm formation; XTT	[37]
	EO: no composition	*C. albicans*	48,000	3300	Biofilm formation; XTT	[38]
*Thymus vulgaris* L. (Lamiaceae)	EO: thymol (54.73%), carvacrol (12.42%), terpineol (4.00%), nerol acetate (2.86%), fenchol (0.5%)	*C. albicans*	1.56–25	12.5	Biofilm formation; absorbance, crystal violet, and scanning electron microscopy	[26]
		*C. tropicalis*	25–50	12.5		
*Warburgia ugandensis* Sprague (Canellaceae)	Extract: ugandenial A, warburganal, polygodial, alpha-linolenic acid ALA	*C. albicans*	Lack of data	1000	Biofilm formation and mature biofilm; XTT and confocal laser microscopy	[52]
		*C. glabrata*	Lack of data	1000		
*Ziziphora tenuior* L. (Lamiaceae)	EO: pulegone (46.8%), p-menth-3-en-8-ol (12.5%), isomenthone (6.6%), 8-hydroxymenthone (6.2%), isomenthol (4.7%)	*C. albicans*	1.25	2.5	Mature biofilm; XTT	[23]
*Zuccagnia punctata* L. (Fabaceae)	Extract: no composition	*C. albicans*	400	100	Biofilm formation and mature biofilm; XTT and crystal violet	[53]

Legend: MIC—minimal inhibitory concentration; XTT—reduction assay of 2,3-bis(2-methoxy-4-nitro-5-sulfophenyl)-5-[carbonyl(phenylamino)]-2H-tetrazolium hydroxide; MTT—reduction assay of 3-(4,5-dimethylthiazol-2-yl)-2,5-diphenyltetrazolium bromide [54,55].

**Table 2:** Antifungal and antibiofilm activity of plant compounds.

Active Compound	Example of Plant Origin	Targeted Fungus	MICs (mg/L, mL/L)	Inhibition of Biofilm Formation by at Least 50% (mg/L, mL/L)	Inhibited Stage of Biofilm; Method of Biofilm Detection	Ref.
Antidesmone (alkaloid)	*Waltheria indica*, *W. brachypetala*	*C. albicans*	32	16	Mature biofilm; XTT	[63]
		*C. glabrata*	>32	16		
		*C. krusei*	16	16		
		*C. parapsilosis*	4	16		
		*C. tropicalis*	>32	16		
Anisaldehyde (phenolic aldehyde)	* Pimpinella anisum * , * Foeniculum vulgare *	*C. albicans*	500	500	Mature biofilm; XTT, crystal violet, and inverted light microscopy	[68]
Anisic acid (phenolic acid)	* Pimpinella anisum *	*C. albicans*	4000	4000	Mature biofilm; XTT, crystal violet, and inverted light microscopy	[68]
Anisyl alcohol (phenolic alcohol)	* Pimpinella anisum *	*C. albicans*	31	500	Mature biofilm; XTT, crystal violet, and inverted light microscopy	[68]
Baicalein (flavonoid)	*Scutellaria baicalensis*, *S. lateriflora*	*C. albicans*	No data	4–32	Biofilm formation; XTT	[62]
Camphene (monotherpene)	*Croton eluteria*, *Cinnamomum verum*	*C. albicans*	No data	500	Biofilm formation; confocal laser microscopy	[36]
		*C. albicans*	1000	2000	Mature biofilm; XTT, crystal violet, and inverted light microscopy	[69]
Camphor (bicyclic monotherpene)	*Cinnamomum camphora*, *Artemisia annua*	*C. albicans*	125–250	Not or 62.5–250	Biofilm formation; crystal violet and absorbance	[70]
		*C. glabrata*	175	Not		
		*C. krusei*	350	Not		
		*C. parapsilosis*	125	Not		
		*C. tropicalis*	175	175		
Cannabidiol (cannabinoid)	*Cannabis sativa*	*C. albicans*	No data	12.5–100	Biofilm formation; confocal microscopy	[66]
Carvacrol (phenol)	*Thymus serpyllum*, *Carum carvi*, *Origanum vulgare*	*C. albicans*	250	500	Mature biofilm; XTT, crystal violet, and inverted light microscopy	[69]
			100–20,000	300–1250	Mature biofilm; XTT	[71]
			1000	750–1500	Biofilm formation; MTT	[72]
		*C. glabrata*	100–20,000	300–1250	Mature biofilm; XTT	[71]
		*C. parapsilosis*	100–20,000	300–1250		
Carvene/Limonene (monotherpene)	*Citrus × aurantium*, *Citrus limon*	*C. albicans*	1000	4000	Mature biofilm; XTT, crystal violet, and inverted light microscopy	[69]
Carvone/Carvol (monotherpene)	*Carum carvi*, *Mentha spicata*	*C. albicans*	>4000	250	Mature biofilm; XTT, crystal violet, and inverted light microscopy	[69]
β-Caryophyllene (sesquiterpene)	*Helichrysum italicum*, *Caryophyllusaromaticus*	*C. albicans*	No data	100–500	Biofilm formation; confocal laser microscopy	[36]
1,4-Cineole (monotherpene)	* Rosmarinus officinalis *, *Thymus vulgaris*	*C. albicans*	>4000	4000	Mature biofilm; XTT, crystal violet, and inverted light microscopy	[69]
1,8-Cineole/Eucalyptol (monotherpene)	*Eucalyptus globulus*, *Salvia officinalis*, *Pinus sylvestris*	*C. albicans*	4000	4000	Mature biofilm; XTT, crystal violet, and inverted light microscopy	[69]
			8	4	Mature biofilm; luminescence	[40]
			3000–23,000	Not or 3000–23,000	Biofilm formation; crystal violet and absorbance	[70]
		*C. glabrata*	2000	Not		
		*C. krusei*	4000	2000–4000		
		*C. parapsilosis*	2000	1000–2000		
		*C. tropicalis*	4000	2000–4000		
Cinnamaldehyde (aldehyde)	*Cinnamomum* sp., *Apium graveolens*	*C. albicans*	62	125	Mature biofilm; XTT, crystal violet, and inverted light microscopy	[68]
			50–400	25–200	Mature biofilm; XTT	[58]
Cinnamic acid (phenolic acid)	* Cinnamomum * sp.	*C. albicans*	2000	4000	Mature biofilm; XTT, crystal violet, and inverted light microscopy	[68]
Citral (monotherpene)	*Melissa officinalis*, *Backhousia citriodora*	*C. albicans*	500	1000	Mature biofilm; XTT, crystal violet, and inverted light microscopy	[69]
Citronellal (monotherpene)	* Cymbopogon citratus * , *Melissa officinalis*	*C. albicans*	500	1000	Mature biofilm; XTT, crystal violet, and inverted light microscopy	[69]
β-Citronellol (monotherpene)	*Melissa officinalis*, *Pelargonium roseum*	*C. albicans*	500	1000	Mature biofilm; XTT, crystal violet, and inverted light microscopy	[69]
Cuminaldehyde (monotherpene)	* Carum carvi * , * Cinnamomum verum *	*C. albicans*	1000 to >4000	6000–7000	Biofilm formation; MTT	[72]
p-Cymene (monotherpene)	*Thymus vulgaris*, *Eucalyptus* sp.	*C. albicans*	2000	4000	Mature biofilm; XTT, crystal violet, and inverted light microscopy	[69]
8-Deoxoantidesmone (alkaloid)	*Waltheria indica*	*C. albicans*	16	32	Mature biofilm; XTT	[63]
		*C. glabrata*	>32	32		
		*C. krusei*	32	32		
		*C. parapsilosis*	32	32		
		*C. tropicalis*	>32	32		
2′,4′-Dihydroxy-3′-methoxychalcone (chalcone)	*Zuccagnia punctata*, *Oxytropis falcata*	*C. albicans*	100	25	Biofilm formation and mature biofilm; XTT and crystal violet	[53]
Dioscin (steroidal saponin)	*Dioscorea* sp., *Chamaecostus*	*C. albicans*	3.9–15.62	3.9–31.25	Biofilm formation and mature biofilm; MTT	[31]
Ellagic acid (polyphenol)	*Punica granatum* L.	*C. albicans*	75–100	25–40	Biofilm formation and mature biofilm; crystal violet	[49]
Emodin (anthraquinone)	*Rheum palmatum*, *Frangula alnus*	*C. albicans*	12.5–50	Not or 100–400	Biofilm adhesion; MTT	[73]
4α,5α-Epoxy-10α,14H-1-epi-inuviscolide (sesquiterpene lactone)	*Carpesium macrocephalum*	*C. albicans*	>128	38	Biofilm formation and mature biofilm; XTT	[67]
Eugenol (phenol)	* Syzygium aromaticum * , * Cinnamomum * sp.	*C. albicans*	50–400	12.5–200	Mature biofilm; XTT	[58]
			250	500	Mature biofilm; XTT, crystal violet, and inverted light microscopy	[69]
			500	500	Mature biofilm; XTT, crystal violet, and inverted light microscopy	[68]
			1200	10,000–80,000	Mature biofilm; XTT	[59]
Farnesol (sesquiterpene)	*Tilia* sp., *Cymbopogon* sp.	*C. albicans*	1000	500	Mature biofilm; XTT, crystal violet, and inverted light microscopy	[68]
			1000	500	Mature biofilm; XTT, crystal violet, and inverted light microscopy	[69]
Gallic acid (phenolic acid)	*Polygonum* sp., *Buchenavia tomentosa*	*C. albicans*	5000	2500	Biofilm formation and mature biofilm; culture	[30]
Geraniol (monotherpene)	*Pelargonium graveolens*, *Rosa* sp.	*C. albicans*	1000	1000	Mature biofilm; XTT, crystal violet, and inverted light microscopy	[69]
		*C. albicans*	100–20,000	300–1250	Mature biofilm; XTT	[71]
		*C. albicans*	No data	1000–8000	Mature biofilm; XTT	[47]
		*C. glabrata*	100–20,000	300–1250	Mature biofilm; XTT	[71]
		*C. parapsilosis*	100–20,000	300–1250		
Guaiacol (phenol)	* Guaiacum officinale * , *Apium graveolens*	*C. albicans*	500	1000	Mature biofilm; XTT, crystal violet, and inverted light microscopy	[68]
Hydroxychavicol (phenol)	*Piper betle*	*C. albicans*	125–500	125–1000	Biofilm formation and mature biofilm; XTT	[74]
β-Ionone (carotenoid)	* Lawsonia inermis * , * Camellia sinensis *	*C. albicans*	250	250	Mature biofilm; XTT, crystal violet, and inverted light microscopy	[69]
Isomontanolide (sesquiterpenic lactone)	*Laserpitium ochridanum*, *L. zernyi*	*C. albicans*	50	250	Mature biofilm; luminescence	[43]
		*C. krusei*	200	250		
Isopulegol (monotherpene)	* Mentha rotundifolia*, *Melissa officinalis*	*C. albicans*	>4000	250	Mature biofilm; XTT, crystal violet, and inverted light microscopy	[69]
Ivalin (sesquiterpene lactone)	*Geigeria aspera*, *Carpesium macrocephalum*	*C. albicans*	>128	15.4	Biofilm formation and mature biofilm; XTT	[67]
Laserpitine (sesquiterpene lactone)	*Laserpitium latifolium*, *Laserpitiumhalleri*	*C. albicans*	200	400	Mature biofilm; luminescence	[43]
		*C. krusei*	200	400		
Lichochalcone A (chalconoid)	*Glycyrrhiza* sp.	*C. albicans*	6.25–12.5	0.2–20	Biofilm formation; crystal violet	[61]
Linalool (monotherpene)	*Lavandula officinalis*, *Pelargonium graveolens*	*C. albicans*	No data	100–500	Biofilm formation; confocal laser microscopy	[36]
			2000	1000	Mature biofilm; XTT, crystal violet, and inverted light microscopy	[69]
			No data	1000–8000	Mature biofilm; XTT	[47]
α-Longipinene (sesquiterpene)	*Croton eluteria*, *Helichrysum italicum*	*C. albicans*	No data	100–500	Biofilm formation; confocal laser microscopy	[36]
Menthol (monotherpene)	*Mentha* spp.	*C. albicans*	>4000	2000	Mature biofilm; XTT, crystal violet, and inverted light microscopy	[69]
			2500	10,000–80,000	Mature biofilm; XTT	[59]
Montanolide (sesquiterpene lactone)	*Laserpitium ochridanum*, *L. zernyi*	*C. albicans*	200	400	Mature biofilm; luminescence	[43]
		*C. krusei*	200	400		
Morin (flavonoid)	* Prunus dulcis * , * Morus alba *	*C. albicans*	150	37.5–600	Biofilm formation; crystal violet	[75]
Myrcene (monotherpene)	*Humulus lupulus*, *Cannabis sativa*	*C. albicans*	1000	2000	Mature biofilm; XTT, crystal violet, and inverted light microscopy	[69]
Nerol (monotherpene)	*Citrus × aurantium*, *Humulus lupulus*	*C. albicans*	2000	500	Mature biofilm; XTT, crystal violet, and inverted light microscopy	[69]
Nerolidols (sesquiterpene)	*Citrus × aurantium*, *Piper claussenianum*	*C. albicans*	18,600–62,500	2500–10,000	Mature biofilm; MTT	[48]
α-Pinene (monotherpene)	*Pinus sylvestris*, *Picea abies*	*C. albicans*	3125	3125	Biofilm formation; XTT	[76]
β-Pinene (monotherpene)	*Pinus sylvestris*, *Picea abies*	*C. albicans*	2000	4000	Mature biofilm; XTT, crystal violet, and inverted light microscopy	[69]
			187	187	Biofilm formation; XTT	[76]
Polygodial (sesquiterpene)	*Warburgia ugandensis*, *Polygonum hydropiper*	*C. albicans*	4.1	10.8	Biofilm formation and mature biofilm; XTT and confocal laser microscopy	[52]
		*C. glabrata*	94.1	50.6–61.9		
Pterostilbene (polyphenol)	*Pterocarpus marsupium*, *Pterocarpus santalinus*, *Vitis vinifera*	*C. albicans*	No data	8–32	Biofilm formation and mature biofilm; XTT	[65]
Riccardin D (macrocyclic bisbibenzyl)	*Dumortiera hirsuta*	*C. albicans*	16	8–64	Mature biofilm; XTT	[64]
Salicylaldehyde (phenolic aldehyde)	*Filipendula ulmaria*, *Fagopyrum esculentum*	*C. albicans*	31	125	Mature biofilm; XTT, crystal violet, and inverted light microscopy	[68]
Salicylic acid (phenolic acid)	*Salix* sp., *Filipendula ulmaria*	*C. albicans*	4000	2000	Mature biofilm; XTT, crystal violet, and inverted light microscopy	[68]
Scopoletin (cumarin)	*Mitracarpus frigidus*, *Scopolia carniola*	*C. tropicalis*	50	50	Biofilm adhesion, formation, and mature biofilm; absorbance and digital scanning	[77]
6-Shogaol (phenylalkane)	*Zingiber officinale*	*C. auris*	32–64	16–64	Mature biofilm; crystal violet	[78]
Tarolide (sesquiterpene lactone)	*Laserpitium ochridanum*, *L. zernyi*	*C. albicans*	400	1000	Mature biofilm; luminescence	[43]
		*C. krusei*	400	1000		
Telekin (sesquiterpene lactone)	*Carpesium macrocephalum*, *Telekia speciose*	*C. albicans*	>128	36	Biofilm formation and mature biofilm; XTT	[67]
Terpinolene (terpene)	*Cannabis sativa*, *Citrus limon*	*C. albicans*	2000	4000	Mature biofilm; XTT, crystal violet, and inverted light microscopy	[69]
5,7,3′,4′-Tetramethoxyflavone (flavonoid)	*Psiadia punctulate*, *Kaempferia parviflora*	*C. albicans*	100	40	Biofilm formation; crystal violet	[79]
α-Thujone (monotherpene)	*Artemisia absinthium*, *Tanacetum vulgare*	*C. albicans*	>4000	500	Mature biofilm; XTT, crystal violet, and inverted light microscopy	[69]
Thymol (phenol)	*Thymus vulgaris*, *Trachyspermum copticum*	*C. albicans*	250	250	Mature biofilm; XTT, crystal violet, and inverted light microscopy	[69]
			1.56–50	3.12	Biofilm formation; absorbance, crystal violet, and scanning electron microscopy	[26]
			32–128	128	Biofilm adhesion and mature biofilm; XTT	[80]
			100–20,000	300–1250	Mature biofilm; XTT	[71]
			125	125–250	Biofilm formation and mature biofilm; XTT	[81]
			1200	5000–80,000	Mature biofilm; XTT	[59]
		*C. tropicalis*	1.56–50	12.5	Biofilm formation; absorbance, crystal violet, and scanning electron microscopy	[26]
		*C. glabrata*	100–20,000	300–1250	Mature biofilm; XTT	[71]
		*C. parapsilosis*	100–20,000	300–1250		
Tn-AFP1 (protein)	*Trapa natans*	*C. tropicalis*	32	16	Mature biofilm; XTT	[82]
5,6,8-Trihydroxy-7,4′ dimethoxy flavone (flavonoid)	*Thymus membranaceus* subsp. *membranaceus*, *Dodonaea viscosa* var. *angustifolia*	*C. albicans*	390	390	Biofilm formation and mature biofilm; MTT	[83]
5(R)-Vanessine (alkaloid)	*Waltheria indica*	*C. albicans*	32	16	Mature biofilm; XTT	[63]
		*C. glabrata*	>32	16		
		*C. krusei*	32	16		
		*C. parapsilosis*	>32	16		
		*C. tropicalis*	>32	16		
Vanillic acid (phenolic acid)	* Angelica sinensis * , *Solanum tuberosum*	*C. albicans*	>4000	4000	Mature biofilm; XTT, crystal violet, and inverted light microscopy	[68]
Vanillin (phenol)	*Vanilla planifolia*	*C. albicans*	1000	500	Mature biofilm; XTT, crystal violet, and inverted light microscopy	[68]
Waltheriones (alkaloid)	*Waltheria indica*, *W.viscosissima*	*C. albicans*	4–32	8–32	Mature biofilm; XTT	[63]
		*C. glabrata*	32 or >32	8–32		
		*C. krusei*	16–32 or >32	8–32		
		*C. parapsilosis*	2–32 or >32	8–32		
		*C. tropicalis*	32 or >32	8–32		
Warburganal (sesquiterpene)	*Warburgia* sp.	*C. albicans*	4	4.5	Biofilm formation and mature biofilm; XTT and confocal laser microscopy	[52]
		*C. glabrata*	72–72.6	49.1–55.9		

Legend: MIC—minimal inhibitory concentration; XTT—reduction assay of 2,3-bis(2-methoxy-4-nitro-5-sulfophenyl)-5-[carbonyl(phenylamino)]-2H-tetrazolium hydroxide; MTT—reduction assay of 3-(4,5-dimethylthiazol-2-yl)-2,5-diphenyltetrazolium bromide [54,55].
